# Rift Valley Fever Virus among Wild Ruminants, Etosha National Park, Namibia, 2011

**DOI:** 10.3201/eid2201.150725

**Published:** 2016-01

**Authors:** Andrea Capobianco Dondona, Ortwin Aschenborn, Chiara Pinoni, Luigina Di Gialleonardo, Adrianatus Maseke, Grazia Bortone, Andrea Polci, Massimo Scacchia, Umberto Molini, Federica Monaco

**Affiliations:** Istituto Zooprofilattico Sperimentale dell’Abruzzo e del Molise “G. Caporale,” Teramo, Italy (A. Capobianco Dondona, C. Pinoni, L. Di Gialleonardo, G. Bortone, A. Polci, M. Scacchia, U. Molini, F. Monaco);; Ministry of Environment and Tourism, Windhoek, Namibia (O. Ashenborn);; Ministry of Agriculture, Water & Forestry, Windhoek (A. Maseke)

**Keywords:** Rift Valley fever, wildlife, epidemiology, Etosha, Namibia, springbok, Antidorcas marsupialis, wildebeest, Connochaetes taurinus, black-faced impala, Aepyceros melampus petersi, viruses, zoonoses

## Abstract

After a May 2011 outbreak of Rift Valley fever among livestock northeast of Etosha National Park, Namibia, wild ruminants in the park were tested for the virus. Antibodies were detected in springbok, wildebeest, and black-faced impala, and viral RNA was detected in springbok. Seroprevalence was high, and immune response was long lasting.

Rift Valley fever virus (RVFV) is a zoonotic mosquito-borne virus that infects humans and a wide range of domestic and wild ruminants. RVFV is a phlebovirus within the family *Bunyaviridae*. The RVFV genome comprises 3 single-stranded RNA segments that encode structural and nonstructural proteins (*1*). Mosquitoes, mainly from the genera *Aedes*, *Culex*, and *Anopheles*, spread the virus between animals and humans (*1*); direct transmission through aerosol spread of infected biological fluids plays a major role in human infection (*2*). RVFV was first isolated in the Rift Valley of Kenya in the early 1930s; since then, multiple epizootics and epidemics among animals and humans have occurred in Africa, Madagascar, the Comoros Archipelago, and the Arabian Peninsula (*3*). Although outbreaks are often underreported because of surveillance deficiencies, RVF is considered endemic to many African countries, where outbreaks occur at irregular intervals, usually after exceptionally heavy rains and floods (*4*).

During May 2011, an outbreak of RVF occurred among livestock in the Oshikoto region of Namibia, northeast of Etosha National Park (*5*). The ongoing sampling of wildlife as part of the surveillance program for Emerging Infectious Diseases and Transboundary Animal Diseases in Etosha National Park provided an opportunity to investigate the role played by wildlife in RVF epidemiology. We report detection of RVFV in springbok (*Antidorcas marsupialis*), wildebeest (*Connochaetes taurinus*), and black-faced impala (*Aepyceros melampus petersi*) in Etosha National Park.

## The Study

To maximize the chances of detecting RVFV circulation in a potentially infected area with extensive mixing of susceptible animals, widespread distribution of vectors, and consequently no particular factors that would lead to sampling bias, we chose to collect samples from animal species with a long life expectancy and widespread distribution. In collaboration with local staff and veterinary authorities, the first phase of sampling was conducted during May–July 2011. During the first phase, 200 springbok and 50 wildebeest were randomly selected, immobilized by darting, and fitted with radio collars for identification. During the second phase (December 2011), 45 springbok, 7 wildebeest, and 8 black-faced impala were sampled. Of these, 15 springbok and 4 wildebeest that had been sampled in phase 1 were recaptured. During both phases, blood samples were collected from each animal for serologic and virologic investigations.

We investigated the presence of antibodies against RVFV in serum samples by using 2 ELISAs (both from ID Vet, Grabels, France): 1) the ID Screen Rift Valley Fever Competition Multi-species Kit, to detect total antibody activity; and 2) the ID Screen Rift Valley Fever IgM Kit, to detect IgM against RVFV. RNA was purified from serum by using the High Pure Viral Nucleic Acid extraction kit (Roche Diagnostics, Mannheim, Germany) in accordance with the manufacturer’s instructions. The presence of RVFV RNA was determined by specific real-time reverse transcription PCR (rRT-PCR) as previously described (*6*) by using primers and probe targeting the large segment of RVFV genome.

During phase 1, antibody activity was detected in 70 (35%) of 200 springbok (95% CI 28.73–41.85) and in 12 (24%) of 50 wildebeest (95% CI 14.33–37.49). IgM was detected in 30 (15%) of 200 springbok (95% CI 10.73–20.62) (Table 1). Viral RNA was detected in 18 (9%) of 200 springbok (95% CI 5.79–13.78). Of these 18 springbok, 7 were seropositive for RVFV and 4 were positive for IgM only (Table 1). Antibodies against RVFV were not detected in the remaining 11 springbok with positive rRT-PCR results. During phase 2, antibody activity was detected in 25 (56%) of 45 springbok (95% CI 41.11–69.10), in 1 (14%) of 7 wildebeest (95% CI 3.19–52.65), and in 5 (63%) of 8 black-faced impalas (95% CI 29.9–86.30) (Table 1). In December 2011, no rRT-PCR or IgM results were positive for any animal, but among the 15 springbok and 4 wildebeest recaptured, RVF antibody activity was detected for 11 animals (10 springbok and 1 wildebeest). In particular, 2 of the 18 viremic springbok that were positive by rRT-PCR during phase 1 showed seroconversion, and a persistent immune response was detected in the 6 of the 70 seropositive animals after resampling 6 months apart (Table 2).

**Table 1:** Results of serologic and virologic testing of wild ruminants for Rift Valley fever virus, Etosha National Park, Namibia, 2011*

Animal and time of sampling	rRT-PCR	IgM	Total antibodies other than IgM	No. positive/no. tested (% positive)
Springbok (*Antidorcas marsupialis*), n = 230				
May–Jul	–	–	–	119/200 (59.5)
	–	–	+	37/200 (18.5)
	–	+	–	26/200 (13.0)
	+	–	–	11/200 (5.5)
	+	+	–	4/200 (2.0)
	+	–	+	3/200 (1.5)
Dec	–	–	–	20/45 (44.4)†
	–	–	+	25/45 (55.6)†
Wildebeest (*Connochaetes taurinus*), n = 53				
May–Jul	–	–	–	38/50 (76.0)
	–	–	+	12/50 (24.0)
Dec	–	–	–	6/7 (85.7)†
	–	–	+	1/7 (14.3)†
Black-faced impala (*Aepyceros melampus petersi*), n = 8				
Dec	–	–	–	3/8 (37.5)
	–	–	+	5/8 (62.5)

*rRT-PCR, real-time reverse transcription PCR. †15 springbok and 4 wildebeest were recaptured during the second phase of sampling (December 2011).

**Table 2:** Evolution of Rift Valley fever virologic and immune status of recaptured animals, Etosha National Park, Namibia, 2011*

Animal	May–Jul				Dec			No. (%) positive/no. tested (% positive)
	rRT-PCR	IgM	Total antibody		rRT-PCR	IgM	Total antibody	
Springbok (*Antidorcas marsupialis*)	–	–	–		–	–	–	5/15 (33.3)
	–	–	–		–	–	+	2/15 (13.3)
	+	–	–		–	–	+	2/15 (13.3)
	–	+	–		–	–	+	1/15 (6.7)
	–	–	+		–	–	+	5/15 (33.3)
Wildebeest (*Connochaetes taurinus*)	–	–	–		–	–	–	3/4 (75.0)
	–	–	–		–	–	+	1/4 (25.0)

*rRT-PCR, real-time reverse transcription PCR.

## Conclusions

After the 2010 epidemic of RVF in Namibia (*7*), 3 outbreaks affecting domestic livestock were reported in the Oshikoto region of northern Namibia (*5*). RVFV circulation in Etosha National Park during May–July 2011 was demonstrated by the capture of viremic (RNA-positive) and recently infected (IgM-positive) springbok. Aware that ELISA results rely on the use of commercial tests not validated for wildlife testing, we supported our serologic findings by the detection of RVFV genome in the serum of springbok and seroconversion of viremic animals 6 months later. Furthermore, the contemporaneous detection of RVFV genome and antibody activity in springbok serum are consistent with data generated by experimental infection of livestock (*2*,*8*,*9*). Our findings also confirmed wildlife capability to develop a long-lasting immune response after RVFV infection (*2**,**10*).

The high seroprevalence among wild ruminants suggests intense RVFV activity in the area during May–July. Active virus circulation was not detected in December 2011, when only antibody activity was detected. It can be assumed that the onset of the dry season, which suppressed vector activity, associated with the high seroprevalence in susceptible hosts, mainly contributed to the abatement of virus circulation.

Although antibodies against RVFV have been detected in many species of wildlife, including springbok, wildebeest, and impala (*11*–*13*), the epidemiologic role of wildlife is far from elucidated. Wildlife are suspected to play a role during interepidemic periods, but their involvement during epidemics and the existence of sylvatic cycles involving wildlife and mosquitoes in maintenance and perpetuation of RVFV has rarely been investigated (*12*). More likely, RVFV persistence results from a balance of transmission between numerous susceptible vertebrates and mosquitoes, including vertical transmission in vectors (*14*). Further investigation of the duration and the extent of RVFV viremia in wild ruminants could therefore clarify the potential role of wildlife in maintaining the disease in the environment. This information could lead to hypotheses of the origin of the virus that circulated in Etosha National Park during 2011 and whether it originated from neighboring areas or arose from an adaptation to wildlife from cryptic endemic foci. 

Whatever the source of infection, RVFV in wildlife has only recently (2008–2011) been associated with severe disease in South Africa, where outbreaks began with abortions in buffaloes and the death of a waterbuck, but infection of livestock was not reported (*10**,**15*). The large number of predators within Etosha National Park may have prevented detection of clinical cases and may explain why no abortions, hemorrhagic disease, or deaths were reported during this study. The lack of human cases in the park or surrounding areas does not imply a lack of threat to public health. Whether the differences in prevalence of viremic animals and in serologic results may be associated with higher resistance of wildebeest to infection with RVFV or to differences in vector exposure should be investigated. More studies are warranted to gain a better understanding of the role of wildlife in the spread of RVFV and the interepidemic maintenance of the virus and to explore the potential use of wild ruminants in Africa as sentinels for monitoring RVFV activity.
